# Menopausal status dependence of the timing of breast cancer recurrence after surgical removal of the primary tumour

**DOI:** 10.1186/bcr937

**Published:** 2004-10-11

**Authors:** Romano Demicheli, Gianni Bonadonna, William JM Hrushesky, Michael W Retsky, Pinuccia Valagussa

**Affiliations:** 1 Istituto Nazionale Tumori, Milano, Italy; 2 Dorn VA Medical Center, Columbia, South Carolina, USA; 3 Department of Surgery, Children's Hospital and Harvard Medical School, Boston, Massachusetts, USA

**Keywords:** breast cancer, menopausal status, metastasis development model, recurrence timing, tumour dormancy

## Abstract

**Introduction:**

Information on the metastasis process in breast cancer patients undergoing primary tumour removal may be extracted from an analysis of the timing of clinical recurrence.

**Methods:**

The hazard rate for local-regional and/or distant recurrence as the first event during the first 4 years after surgery was studied in 1173 patients undergoing mastectomy alone as primary treatment for operable breast cancer. Subset analyses were performed according to tumour size, axillary nodal status and menopausal status.

**Results:**

A sharp two-peaked hazard function was observed for node-positive pre-menopausal patients, whereas results from node-positive post-menopausal women always displayed a single broad peak. The first narrow peak among pre-menopausal women showed a very steep rise to a maximum about 8–10 months after mastectomy. The second peak was considerably broader, reaching its maximum at 28–30 months. Post-menopausal patients displayed a wide, nearly symmetrical peak with maximum risk at about 18–20 months. Peaks displayed increasing height with increasing axillary lymph node involvement. No multi-peaked pattern was evident for either pre-menopausal or post-menopausal node-negative patients; however, this finding should be considered cautiously because of the limited number of events. Tumour size influenced recurrence risk but not its timing. Findings resulting from the different subsets of patients were remarkably coherent and each observed peak maintained the same position on the time axis in all analysed subsets.

**Conclusions:**

The risk of early recurrence for node positive patients is dependent on menopausal status. The amount of axillary nodal involvement and the tumour size modulate the risk value at any given time. For pre-menopausal node-positive patients, the abrupt increase of the first narrow peak of the recurrence risk suggests a triggering event that synchronises early risk. We suggest that this event is the surgical removal of the primary tumour. The later, broader, more symmetrical risk peaks indicate that some features of the corresponding metastatic development may present stochastic traits. A metastasis development model incorporating tumour dormancy in specific micro-metastatic phases, stochastic transitions between them and sudden acceleration of the metastatic process by surgery can explain these risk dynamics.

## Introduction

The 1970s and 1980s witnessed a revolution in the conventional approach to the treatment of primary breast cancer. Early in the 1970s, the favourable results of postoperative systemic adjuvant therapy in women with positive axillary lymph nodes [1,2] started an avalanche of clinical trials that explored the role of several systemic treatments in different subsets of patients. The beneficial results were confirmed by a few overviews of randomised trials [3,4], and reports from individual studies proved that the benefit continued at 20 years of follow-up [5]. However, the significant, albeit moderate, improvement of disease-free survival and overall survival achieved by earlier adjuvant therapy trials has improved only slightly during subsequent years, despite a spate of new active drugs and the use of higher drug doses [6]. The benefits of adjuvant therapy have therefore apparently reached a plateau, and it is unlikely that further improvements will be obtained without a more complete and accurate understanding of the biology of the tumour–host system at the time of treatment.

Surgical resection of primary tumour removal may either 'cure' a significant fraction of patients, or it may even change the 'natural' recurrence and death timing for some others, by accelerating the metastatic development [7,8]. Some specific biological mechanisms supporting this effect have been elucidated: in animals given surgery, a growth-stimulating factor was found in serum [9] and a switch of micro-metastatic foci to the angiogenic phenotype, due to withdrawal of an angiogenesis inhibitor from the primary tumour, was demonstrated [10]. Despite these provocative data, the residual tumour growth dynamics underlying the beneficial results of all adjuvant systemic treatments is virtually unknown in humans.

Careful inspection of the timing of tumour recurrence after resection can be of considerable interest. The recurrence risk pattern in a given follow-up span, a useful estimate of which is the hazard function [11], provided information on the biological behaviour of metastases. The hazard functions for local-regional recurrences and distant metastases for breast cancer patients undergoing mastectomy alone [12] proved to be double-peaked, with an early peak at about 18 months after surgery, a second peak at about 60 months, and a plateau-like tail extending out to 15 years, the maximum period analysed. These findings were confirmed by a similar investigation on node-positive patients receiving adjuvant CMF (cyclophosphamide, methotrexate, fluorouracil) treatment [13], and a double-peaked hazard function was also demonstrated for the timing of death after primary tumour resection [14-16]. A reasonable hypothesis to explain these findings is that the early peak of the hazard function for recurrence is generated by the sudden acceleration of the metastatic process due to surgery. This hypothesis was the cornerstone for a biological model for the development of breast cancer metastasis, incorporating tumour dormancy in specific micro-metastatic phases and stochastic transitions between them [17]. A computer simulation of the model generated double-peaked relapse histograms reasonably similar to clinical data [18].

The model and the results of its computer simulation suggest that the first peak may result from the superimposition of metastatic recurrences with different dynamics. Here we report a detailed analysis of the first peak of the hazard rate curve, that is, of the recurrence timing during the first 4 years, for patients undergoing mastectomy without any adjuvant chemotherapy or radiotherapy. Our findings suggest that metastatic growth dynamics after primary tumour removal is markedly different depending on menopausal status and, perhaps, axillary nodal involvement. These results support the concept that surgery may have differential perturbing effects on tumour growth kinetics depending on both tumour and host traits. These results strengthen the leading lines of the proposed model for metastasis development and may be relevant to adjuvant treatment strategies.

## Methods

All patients with mastectomy alone as primary treatment who entered into three different clinical trials for operable breast cancer were retrospectively evaluated for this analysis. The trials were performed at the Milan Cancer Institute between 1964 and 1980 to compare mastectomy with other surgical or combined therapeutic approaches. Before surgery, all patients underwent standard staging: complete physical examination, X-ray study of chest, skull, spine and pelvis, bilateral mammography, electrocardiogram, complete haemogram and routine biochemical tests. The primary tumour was treated by radical or modified radical mastectomy. No patient received postoperative radiotherapy or chemotherapy. After surgery, follow-up was performed as follows: physical examination, biochemical tests and chest X-ray every 6–8 months during the first 3 years and once a year thereafter; skeletal survey and mammography once a year. In the presence of controversial clinical findings, examinations were performed more often than originally planned. Appropriate radiological, radioisotopic and surgical investigations were performed whenever recurrence was suspected or clinically evident. Particular attention was paid to assessing the recurrence time by carefully reviewing clinical, radiological and laboratory documentation. More detailed characteristics of the studied series are reported in reference 12.

Treatment failure (recurrence) was defined as the first clinically documented evidence of new disease manifestation(s) in either local-regional area(s) (namely chest wall, axilla and/or ipsilateral supra-clavicular region) or distant site(s) or any combination of these sites (classified as distant). Contra-lateral breast lesions may be either second primaries (confounding factors in the present investigation) or true metastases. Their annual incidence, together with the absence of a link with clinical prognostic factors of the primary breast cancer, suggest that most of them can be considered as second primary breast cancers [12,19]. Contra-lateral tumours were therefore considered second primaries.

Recurrence-free survival was considered as the time elapsed from the date of surgery to the first documented evidence of treatment failure; clinical evaluation without recurrence, death without recurrence, and second primary cancer (including contra-lateral breast cancer) were considered censored events.

Our application involved dividing the time axis into discrete intervals; the timing of the recurrence risk was studied by estimating the recurrence hazard rate, namely the conditional probability of manifesting recurrence in a time interval, given that the patient is clinically free of any recurrence at the beginning of the interval. The discrete hazards of recurrence and their standard deviations were calculated by means of the life-table method over 4 years. Because the event-specific rates display some instability arising from random variation, a kernel-like smoothing procedure [20] was adopted to aid the reader in visualising the underlying pattern, and the smoothed curves were represented graphically. Time intervals of 1, 2, 3, 6 and 12 months were used in a preliminary smoothing analysis that showed that a 3-month interval is a good compromise between smoothing data and displaying structure. We therefore used the hazard rate per 3-month interval.

Menopausal status, which was collected and recorded at the time of primary tumour diagnosis, was defined as 'post-menopausal' if 1 year had elapsed since the last menstrual period. Cumulative survival curves were compared by two-sided log-rank test.

## Results

The main clinical characteristics of the 1173 analysed patients, all undergoing regular follow-up examinations in accordance with protocol requirements, are reported in Table 1. A total of 368 patients suffered from treatment failure within 4 years of surgery: the tumour recurrence was local-regional in 95 cases and distant in 273. A total of 749 patients were surviving and were at risk of recurrence at the end of the fourth year. Patients were lost to follow-up at a rate of 1.6% during the analysed years.

**Figure 1:**
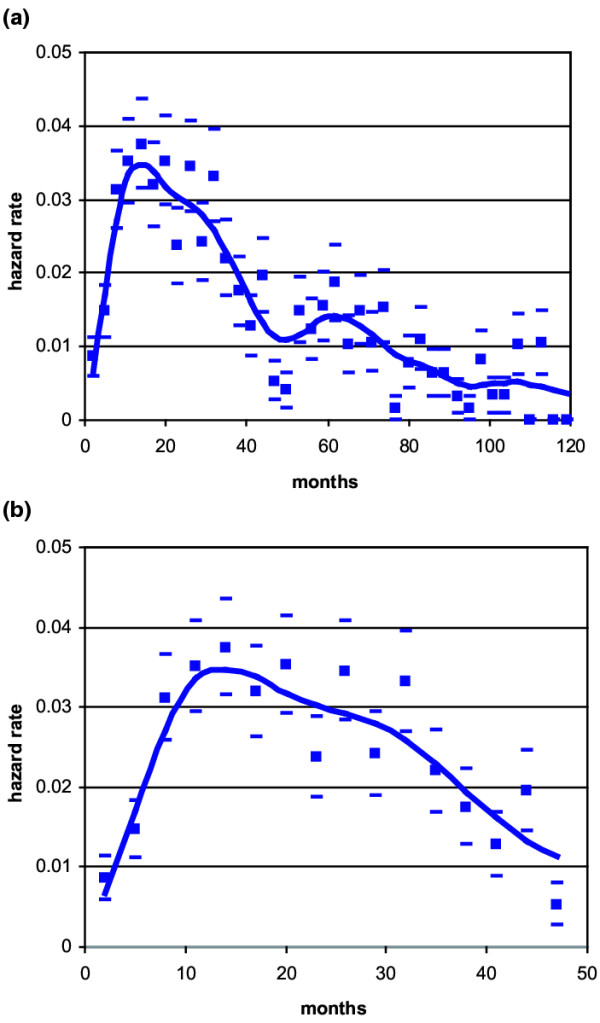
Hazard rate for breast cancer recurrence (local-regional plus distant) after mastectomy alone as primary treatment in 1173 patients. Calculated values (squares) with standard deviations (oblongs) and interpolation curve covering 10 years of follow-up **(a) **and magnification of the first 4 years **(b)**.

As reported and discussed elsewhere [12], the hazard rate for recurrence of all patients during 10 years of follow-up (Fig. 1a) displays a double-peaked pattern. In the present study we focused on the first peak (Fig. 1b), which shows an asymmetrical pattern with an early steep rise, reaching the maximum recurrence risk at the end of the first year, followed by a slight decrease lasting about 1.5 years and then by a more distinct decline until the end of the fourth year. This shape suggests that the recurrence risk curve may result from the superimposition of differently timed peaks.

**Figure 2:**
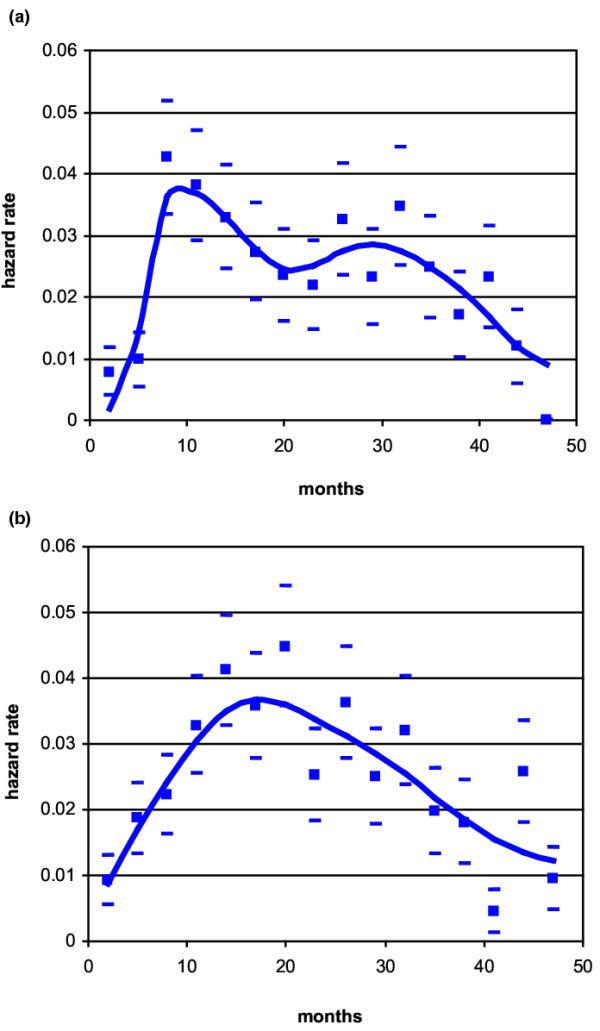
Hazard rate for breast cancer recurrence (local-regional plus distant) after mastectomy alone as primary treatment in 1173 patients: **(a) **pre-menopausal patients; **(b) **post-menopausal patients. Calculated values (squares) with standard deviations (oblongs) and interpolation curve covering the first 4 years of follow-up.

As a first step, the hazard rate pattern by type of recurrence (local-regional versus distant) was studied and, because their timing proved to be remarkably similar, in the further analysis both recurrences were combined. Patients were then allocated to different subsets according to tumour size (T1 versus T2–T3) or to axillary nodal status (positive versus negative) or to menopausal status (pre-menopausal versus post-menopausal). The resulting hazard rate curves indicated that the recurrence risk pattern is definitely correlated to menopausal status, with a double-peaked curve for pre-menopausal patients (Fig. 2a) and a single-peaked curve for post-menopausal women (Fig. 2b). Tumour size did not show a noticeable influence on the recurrence risk pattern, and this finding was confirmed by the analysis of further subsets of patients (T1 and T2–T3 N-pre-menopausal, T1 and T2–T3 N+ pre-menopausal, T1 and T2–T3 N- post-menopausal, T1 and T2–T3 N+ post-menopausal), in whom tumour size (as well as type of recurrence) failed to change the hazard rate pattern (data not shown).

**Figure 3:**
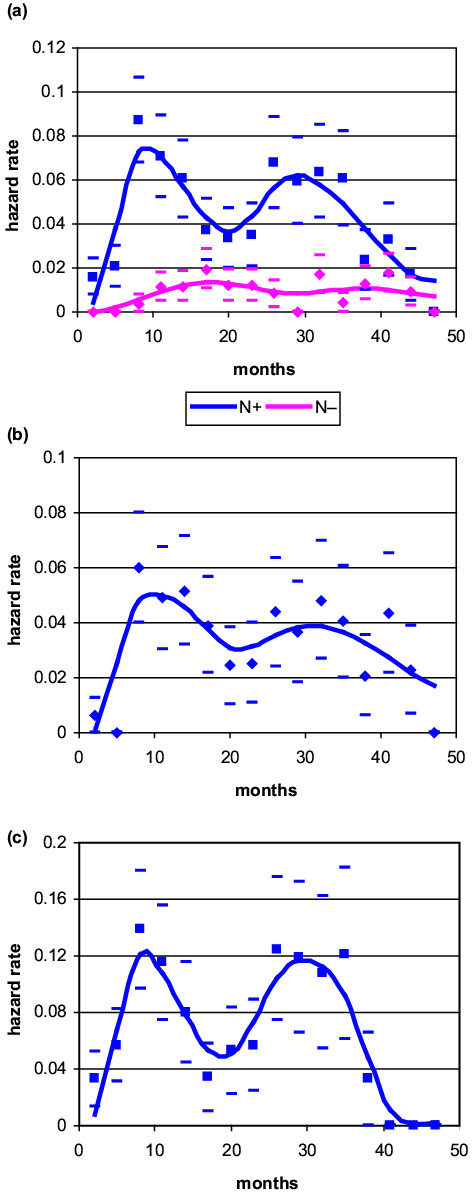
Hazard rate for breast cancer recurrence (local-regional plus distant) after mastectomy alone as primary treatment in pre-menopausal patients: **(a) **node-negative versus node-positive; **(b) **node-positive (one to three) lymph nodes; **(c) **node-positive (more than three) lymph nodes. Calculated values (squares and diamonds) with standard deviations (oblongs) and interpolation curve covering the first 4 years of follow-up (N-, 34 events; N+(1–3), 61 events; N+(>3), 60 events).

Pre-menopausal patients were allocated to subsets of node-negative, node-positive, one to three nodes positive and more than three nodes positive, and the hazard rates were estimated (Fig. 3). The resulting curves show that for node-positive patients, the recurrence risk has a quite narrow early peak, with a very steep rise to a maximum at about 8–10 months after mastectomy, and a second wider increase peaking at about 28–30 months. The recurrence pattern and the peak position do not change for patients with different axillary involvement, whereas the peak height is positively correlated with the extent of nodal invasion.

**Figure 4:**
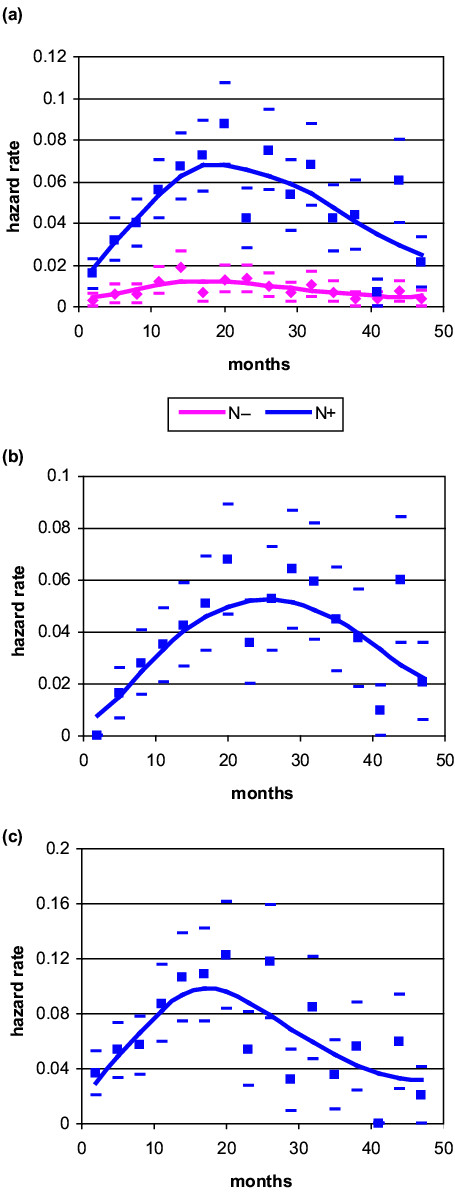
Hazard rate for breast cancer recurrence (local-regional plus distant) after mastectomy alone as primary treatment in post-menopausal patients: **(a) **node-negative versus node-positive; **(b) **node-positive (one to three) lymph nodes; **(c)**, node-positive (more than three) lymph nodes. Calculated values (squares and diamonds) with standard deviations (oblongs) and interpolation curve covering the first 4 years of follow-up (N-, 40 events; N+(1-3), 84 events; N+(>3), 88 events).

A similar analysis was performed in post-menopausal patients. All subsets of patients displayed a wide, nearly symmetrical recurrence risk peak, with a maximum at 18–24 months and a height similar to the corresponding estimates of pre-menopausal patients (Fig. 4). Even for these patients the extent of axillary node invasion was positively correlated to the peak height.

The recurrence risk for node-negative patients displays an initial regular increase reaching a maximum at about 18–24 months after surgery, with a mild decrease afterwards. No multi-peaked pattern was evident or even suggested for either pre-menopausal (Fig. 3a) or post-menopausal (Fig. 4a) women. However, it should be noted that of the 598 node-negative patients at risk, only 74 recurrences were observed during the 4 years under investigation, 43 of which occurred within 2 years. The reported results, mainly those from the third and subsequent years, should therefore be very cautiously considered.

## Discussion

First of all, it should be emphasised that findings resulting from the different subsets of patients are notably coherent. A remarkably stable two-peaked hazard function, with increasing peak height associated with the increasing axillary lymph node involvement, describes the risk of recurrence for pre-menopausal patients, whereas, quite differently, post-menopausal women always display a single peak. Moreover, each observed peak maintains the same position on the time axis in all analysed subsets of pre-menopausal and post-menopausal women. The occurrence of these peaks by chance, that is, resulting from random fluctuations of the recurrence timing, should therefore be considered very unlikely. Obviously, our findings must be confirmed by similar analyses of other comparable databases.

We add here some important details to the previously reported investigation on the recurrence risk pattern of this same series [12]. Indeed, we found that even though pre-menopausal and post-menopausal patients display similar long time results (10-year recurrence-free survival: 56% versus 54%; not significant) and similar overall recurrence pattern (early surge during the first 4 years, a second minor increase at about 5 years and a tapering phase afterwards), the early recurrence dynamics may be very different between pre-menopausal and post-menopausal women.

A very early significant difference in the recurrence risk between pre-menopausal and post-menopausal patients had already been noted [21]. In the present analysis we found that these differences cover all the first 4 years and persist in all patient subsets. Summarising, the recurrence risk for pre-menopausal patients displays an abrupt increase at the third 3-month period; it quickly reaches its prominent maximum level (about four times the level of the previous 6 months) and is followed by a second fairly symmetrical and considerably broader peak appearing at about 18 months of follow-up, with its maximum at 28–30 months after surgery. In comparison, the recurrence risk for post-menopausal patients is nearly symmetrical and, after a smooth increase, reaches its maximum level at about 18 months after primary tumour removal. These findings are clear in node-positive patients, and the higher the nodal involvement, the higher the amplitude of peaks. The limited number of events prevents us from drawing any conclusion for node-negative patients. Tumour size affects the recurrence risk value at a given time but does not affect the pattern of the hazard rate distribution (that is, the occurrence of peaks).

It is difficult not to be impressed by the particular shape of the first recurrence peak of pre-menopausal node-positive patients and by the difference between it and the others of both menopausal subsets. It is also difficult, in the presence of a such an abrupt and prominent increase of the recurrence risk, to imagine something different from a triggering event resulting in recurrence synchronisation.

As a first step, some possible non-biology-based explanations of this finding should be considered. The peak could be related to inadequate staging evaluation, because at the time of the study no bone scan, liver ultrasound or computed tomography scan staging was done. Patients with undiscovered metastatic disease at diagnosis would therefore have displayed recurrence at the initial post-treatment screening, thus contributing to the first peak. However, it should be considered that, for newly diagnosed otherwise asymptomatic operable patients, (1) very few cases prove to be metastatic at standard staging today (less than 5%) [22] and (2) bone scan ultrasound and computed tomography scan surveys have a very low detection rate (less than 1%) [23,24] and are not routinely recommended in these patients [25]. Even chest X-ray was considered unnecessary at staging of asymptomatic operable breast cancer, because of its very low diagnostic yield (0.1%) [26]. Therefore, even if a few missed metastatic patients at primary tumour diagnosis might not have been excluded, they would have had little influence on the observed first peak of recurrence risk involving, for pre-menopausal node-positive patients, 22% of eligible patients and 37% of recurrences within 10 years. Moreover, because up to 75% of breast cancer recurrences are detected by the patient and reported to the physician within a mean time of 1 month [27,28], we can reliably consider the observed recurrence timing as widely independent of the planned follow-up timing. Last but not least, post-menopausal patients, who underwent the same clinical management as pre-menopausal ones, did not display an early peak. A significant role of screening and diagnostics can therefore reasonably be ruled out and a biology-based explanation should be investigated.

The first sharp peak, in our opinion, has a biological explanation and may result from some triggering event changing the unperturbed history of the disease. We suggest that primary tumour surgical removal is probably this event, selectively operating on some micro-metastases within similar biological conditions. This triggering effect could be modulated by the surgery extent [29]. However, in the present investigation data about the different surgical approaches (namely radical versus modified radical mastectomy) were not considered suitable for such an analysis, which would be more usefully performed for trials comparing mastectomy with breast-conserving surgery. The likely triggering effect of mastectomy is also supported by the peak position, implying a rapid activation and growth within 8–10 months. This is in quite good agreement with estimates of tumour growth after dormancy release (30 ± 8 weeks or less) obtained by very different methodology [30].

Assuming tumour dormancy release and the estimate of 8–10 months or less for tumour development from micro-metastasis to clinical metastasis, the hazard function should be considered as a description of the dormancy escape kinetics. A symmetrical, bell-shaped peak in the curve of hazard rate against time provides information about both the mean value (the peak position) and the variance (the peak width) of the timing of the constituent recurrences. The temporally stable position of the recurrence peak therefore suggests a common cause for recurrence development, a narrow peak indicates that the underlying event is most probably deterministic in nature, and a considerably wider peak suggests that some features of the transition from dormancy to growth may also present stochastic traits.

We recently proposed a model [17,18,31] in which breast cancer metastasis development may include successive steps: first, single cells (or nests containing a few cells), where most of malignant cells are non-dividing; second, non-angiogenic micro-metastases (and angiogenic ones in the presence of anti-angiogenic factors) that cannot grow more than the size of avascular foci; and third, vascularised metastases that will reach the clinical level. This orderly process is apparently perturbed by surgical removal of the primary tumour, which can stimulate cells to proliferate and/or remove angiogenic inhibition, thus resulting, for some patients, in sudden acceleration of the metastatic process.

The proposed model both reasonably explains and takes support from the findings of this study. The early sharp peak of the recurrence risk for pre-menopausal patients can be ascribed to a considerable switching of micro-metastatic foci to the angiogenic phenotype via the withdrawal of angiogenesis inhibitor(s) [10], and the sharpness of this peak supports the deterministic character of the triggering event. For post-menopausal patients, for whom the first sharp peak is absent, this effect may be much more modest. Other broader peaks may result from the induced proliferation of single cells through one or more growth-stimulating factors [9], resulting in successive avascular micrometastases, followed by the 'spontaneous' switching to the angiogenic phenotype, a process with stochastic characteristics, in keeping with peak shapes.

As regards differences between pre-menopausal and post-menopausal patients, it should be taken into account that peculiar conditions relevant to breast cancer development, in particular conditions acting on angiogenetic traits, may be dependent on the host hormone milieu. Angiogenesis in invasive breast carcinoma, as evaluated by micro-vessel counts, is associated inversely with patient age, and higher vascular intensity is observed in younger patients [32,33]. Vascular endothelial growth factor, the first member of a family of glycoproteins with considerable stimulating activity for angiogenesis and lymphangiogenesis, waxes and wanes in normal breast tissue within each menstrual cycle [34]. Angiogenin, another potent angiogenic factor, displays a significant difference between serum levels drawn in the proliferative and secretory phases of the menstrual cycle [35]. Plasma levels of endostatin, a negative regulator of angiogenesis, are significantly higher in post-menopausal patients [36]. It is therefore reasonable to assume that some conditions related to menopausal status may select neoplastic cell populations with peculiar traits relative to angiogenesis. A further factor may have a role: it has been suggested that timing of surgery in the menstrual cycle might modulate the tumour–host relationship and ultimately the disease outcome [37]. This occurrence is restricted, obviously, to pre-menopausal patients.

The findings of this investigation would be much more complete if other prognostic and predictive factors could be analysed. However, this database, like most databases of patients undergoing surgery alone without any adjuvant treatment, goes back to years when factors other than TNM (tumor, nodes, metastases) staging and menopausal status were poorly known or completely unknown.

## Conclusions

Early metastatic growth dynamics shows a structured pattern that is correlated to menopausal status. Indeed, during the first 4 years, node-positive pre-menopausal patients display a split of the hazard function surge into two peaks, while post-menopausal patients always display a single peak. The timing and shape of peaks suggest the occurrence of some triggering event, correlated with primary tumour surgical removal, resulting in recurrence synchronisation, which seems to be especially important for pre-menopausal node-positive patients. In addition, findings support the hypothesis that some features of metastatic development may present stochastic traits. A metastasis development model incorporating tumour dormancy, stochastic transitions between specific micro-metastatic phases and sudden acceleration of the metastatic process due to surgery may be considered appropriate and to fit these findings well.

The different post-resection relapse dynamics between pre-menopausal and post-menopausal patients may have important implications for adjuvant systemic treatments. For adjuvant chemotherapy the proposed picture makes it easier to understand retrospectively why adjuvant chemotherapy was found to be most effective in pre-menopausal patients, who during the first months after primary tumour removal would display an enhancement of actively proliferating, and hence more chemo-sensitive, micro-metastases [38]. Most importantly, the prominent role of angiogenesis in the surgery-driven dormancy escape for pre-menopausal patients suggests that early (preoperative) antiangiogenic treatment might favourably influence prognosis.

## Competing interests

The authors declare that they have no competing interests.

## Figures and Tables

**Table 1:** Main patient characteristics

Parameter	Value
Total number	1173
Median age, years (range)	52 (23–82)
Pre-menopausal	517
Tumour size	
T1	222
T2	265
T3	30
Axillary nodes	
N-	266
N+(1–3)	158
N+(>3)	93
Post-menopausal	656
Tumour size	
T1	237
T2	363
T3	56
Axillary nodes	
N-	332
N+(1–3)	184
N+(>3)	140
